# The complete genome sequence of a poplar, *Populus davidiana*

**DOI:** 10.1093/hr/uhag208

**Published:** 2026-06-01

**Authors:** Song Chen, Xinyu Wang, Yunxia Wei, Qiwen Guo, Zhanchen Ren, Yue Yu, Meiqi Lu, Wenxuan Liu, Yuanfu Liu, Siyuan Li, Kanglei Li, Dun Ao, Guanzheng Qu, Xingguo Lan, Wenquan Bao, Su Chen

**Affiliations:** State Key Laboratory of Tree Genetics and Breeding, Northeast Forestry University, Harbin 150040, China; State Key Laboratory of Tree Genetics and Breeding, Northeast Forestry University, Harbin 150040, China; State Key Laboratory of Tree Genetics and Breeding, Northeast Forestry University, Harbin 150040, China; Institute of Forest Ecology, Department of Ecosystem Management, Climate and Biodiversity, University of Natural Resources and Life Sciences, Vienna 1190, Austria; State Key Laboratory of Tree Genetics and Breeding, Northeast Forestry University, Harbin 150040, China; State Key Laboratory of Tree Genetics and Breeding, Northeast Forestry University, Harbin 150040, China; State Key Laboratory of Tree Genetics and Breeding, Northeast Forestry University, Harbin 150040, China; State Key Laboratory of Tree Genetics and Breeding, Northeast Forestry University, Harbin 150040, China; State Key Laboratory of Tree Genetics and Breeding, Northeast Forestry University, Harbin 150040, China; State Key Laboratory of Tree Genetics and Breeding, Northeast Forestry University, Harbin 150040, China; State Key Laboratory of Tree Genetics and Breeding, Northeast Forestry University, Harbin 150040, China; College of Forestry, Inner Mongolia Agricultural University, Hohhot 010019, China; State Key Laboratory of Tree Genetics and Breeding, Northeast Forestry University, Harbin 150040, China; State Key Laboratory of Tree Genetics and Breeding, Northeast Forestry University, Harbin 150040, China; College of Forestry, Inner Mongolia Agricultural University, Hohhot 010019, China; State Key Laboratory of Tree Genetics and Breeding, Northeast Forestry University, Harbin 150040, China

Dear Editor,

The first release of the *Populus trichocarpa* genome reference was achieved by the Joint Genome Institute (JGI) in 2006 [1]. To date, genome assemblies representing at least 21 *Populus* species have been published (Fig. 1d) [2]. With the advances in sequencing technology, poplar assemblies claiming to be gapless or near telomere-to-telomere (T2T) have been reported [3–6]. However, specific genomic regions such as satellite arrays and short arms of acrocentric chromosomes remain unresolved in current assemblies. The short arm of Chromosome 14 (Chr 14) in poplar is particularly intractable due to highly repetitive 45S ribosomal DNA (rDNA) arrays adjacent to telomeres. No *Populus* assembly has yet fully resolved these loci. Quality metrics reveal a widespread presence of spurious *k*-mers (Merqury QV, <86) and low-coverage regions (GCI score, <67) across existing assemblies. Such artifactual and structural errors may arise during the scaffolding of fragmented contigs into chromosome-scale assemblies using genetic maps or Hi-C data. Gap closing can also introduce secondary errors at contig junctions.

**Figure 1:**
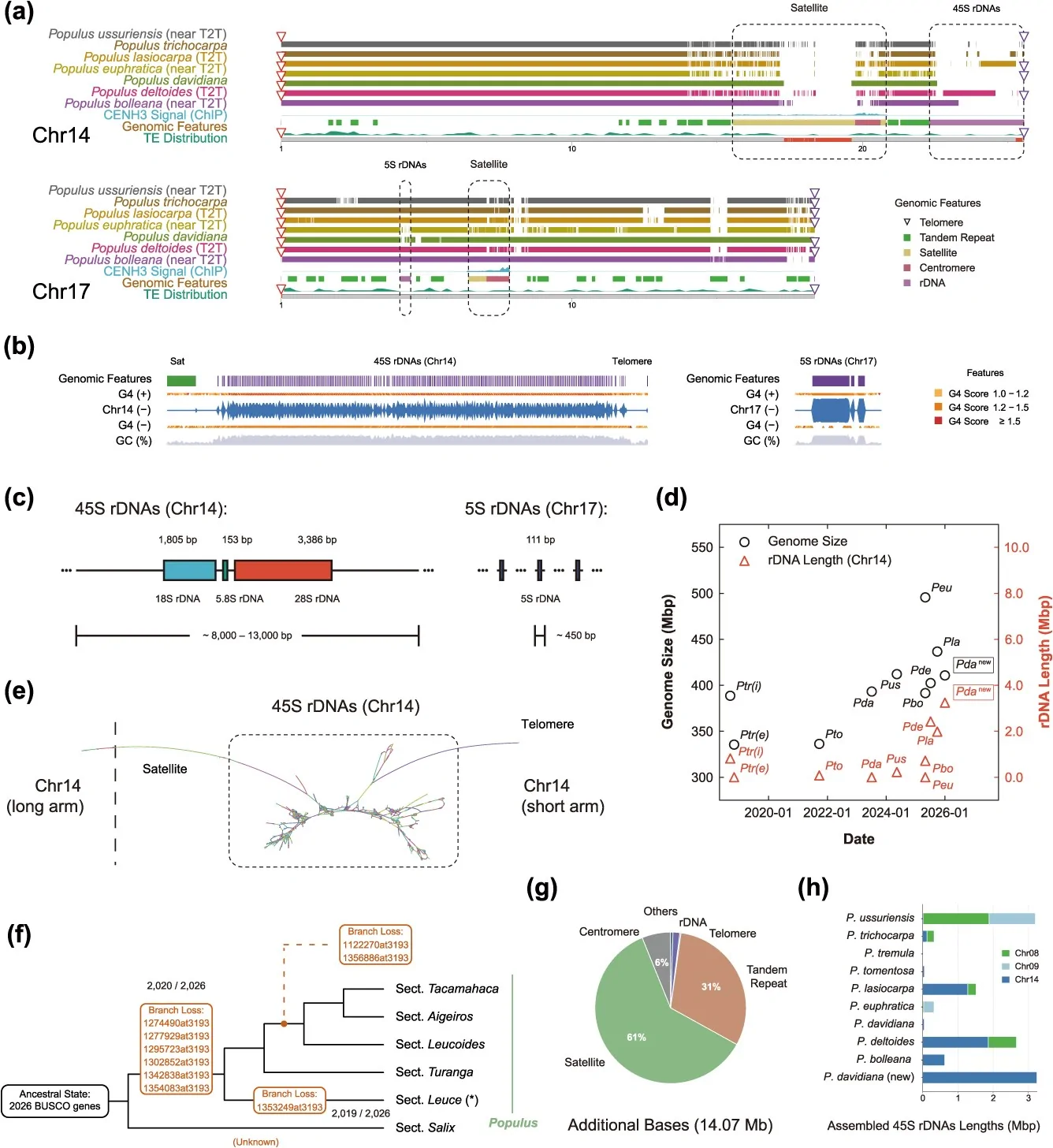
Summary of the complete *P. davidiana* genome assembly. **(a)** Chromosomal ideograms of Chr14 and Chr17, showing TE distribution, genomic features, CENH3 signals, and synteny with previous assemblies. Red marks on the bottom track indicate resolved gaps and corrected assembly errors. Triangles denote telomeres. Unit: Mbp. **(b)** Magnified views of the Chr14 and Chr17 rDNA arrays, showing G-quadruplex (G4) scores on the plus and minus strands, GC content, and the positions of individual rDNA units. **(c)** Canonical repeat units of the Chr14 45S and Chr17 5S rDNA arrays. **(d)** Comparison of assembly length with previous references, shown for the whole genome (circles) and the Chr14 rDNA array (triangles). **(e)** Sparse *de Bruijn* graph of the Chr14 45S rDNA region. Nodes represent sequence segments and edges represent overlaps between them as inferred by the assembler. **(f)** BUSCO gene loss events during the divergence of *Populus* lineages. The phylogeny of *Populus* sections was inferred from 2026 single-copy Embryophyta orthologs (BUSCO odb12), with *Salix* as the outgroup. **(g)** Sequence composition of 14.07 Mbp of newly resolved sequence absent from all prior poplar assemblies. **(h)** Chromosomal location and total length of assembled 45S rDNA loci across poplar assemblies, colored by chromosome of origin (Chr8, Chr9, and Chr14).

To address this challenge, we generated approximately 255× PacBio High-Fidelity (HiFi) reads and 301× Oxford Nanopore Technologies (ONT) long reads of *Populus davidiana* (Table S1, Zenodo). The ONT dataset included ~57× ultra-long reads, which are critical for resolving highly complex repetitive regions. We optimized our assembly workflows to reconstruct highly repetitive centromeric satellite arrays, segmental duplications, and rDNA arrays (Fig. S1, Zenodo). We also generated ~125× Hi-C data to validate the assembly's global structural integrity. Following an evaluation of 14 assembly strategies, we utilized all HiFi reads to construct a string graph using Hifiasm (Table S2, Zenodo). We resolved most graph tangles by estimating coverage depth and node multiplicity to identify unique chromosomal paths. In ambiguous regions, particularly those with recent satellite duplications, we aligned uncorrected ultra-long ONT reads to candidate paths or directly to the HiFi graph to determine the correct traversal. This strategy yielded continuous sequences for 18 of the 19 chromosomes, the only exception was the short arm of Chr 14, where the 45S rDNA arrays and flanking sequences formed the most topologically complex region of the *P. davidiana* genome graph. This locus consists of 13 kb near-identical 45S rRNA repeats arranged in large tandem arrays, spanning approximately 4 Mbp. These arrays could not be resolved using standard Hifiasm string graphs. We therefore employed a specialized approach using sparse *de Bruijn* graphs and ultra-long ONT reads to disentangle the specific repeat variants.

First, we extracted all reads containing rDNA fragments using Barrnap, yielding 9.72 Gb of HiFi and 8.33 Gb of ONT data. To optimize the dataset for assembly, we generated a series of downsampled subsets using Filtlong with a decrement of 0.5 Gb of HiFi and 1.0 Gb of ONT reads. We assembled all pairwise combinations of HiFi and ONT reads using Verkko (Table S3, Zenodo). The optimal graph structure was achieved when using 9.5 Gb of HiFi and 2.0 Gb of ONT reads. We manually resolved the assembly graph tangles using BandageNG and validated junction accuracy via Merqury *k*-mer analysis. This region consists primarily of a continuous 3.24-Mbp 45S rDNA array, including 216 tandem copies of a near-identical 13-kb rRNA repeat unit. Previous assemblies of *Populus deltoides* [5] and *Populus lasiocarpa* [3] captured only 1–2 Mbp of this locus, leaving its flanking architecture unclear. Our complete assembly reveals that the 45S rDNA extends uninterrupted from the pericentromeric boundary to the telomere, spanning most of the short arm of Chr 14. This architecture is consistent with the preferential localization of 45S rDNA on the short arms of acrocentric chromosomes observed across angiosperms.

Finally, we present a gap-free, telomere-to-telomere (T2T) assembly of the *P. davidiana* genome by resolving all centromeric satellite arrays, recent segmental duplications, and the short arms of two acrocentric chromosomes (Chr 9 and 14) (Fig. 1). In total, 14.07 Mbp of sequence absent from all prior poplar assemblies was recovered, which is composed of 60.84% satellites, 30.83% non-satellite segmental duplications, 6.13% centromeres, and 1.46% rDNA (Fig. 1g). We assembled a continuous 3.24-Mbp 45S rDNA array located on the short arm of Chr 14 (Fig. 1h), which was not resolved in the previously published assemblies. Assembly validation confirms exceptional accuracy and consistency (Table S4, Zenodo). HiFi read mapping yielded a Genome Continuity Inspector (GCI) score of 99.9999, and Merqury analysis estimated a Quality Value (QV) approaching infinity, indicating no detectable *k*-mer errors in the assembly. Telomeric repeats capped all 19 chromosomes (range: 448–3083 repeats; mean: 1143.16), confirming a gap-free assembly.

We used TRASH and QuarTeT to annotate the centromeric satellite array on each chromosome (Table S5, Zenodo). The arrays identified in our assembly were longer than those in previous references for most chromosomes. Their relative chromosomal positions were highly consistent across different *Populus* assemblies, supporting the conservation of functional centromere loci across the *Populus* genus (Fig. S2, Zenodo). Given that satellite arrays are typically larger than functional centromeric regions, we generated transgenic lines expressing *FLAG*-tagged endogenous *CENH3* to define functional centromeres using ChIP-seq (Fig. 1a). These CENH3 binding elements span a total of 13.69 Mbp, ranging from 374.75 kbp to 1.11 Mbp (average: 720.62 kbp). Each of the 19 chromosomes contains a single CENH3-enriched region, confirming a monocentric organization in *P. davidiana*. All CENH3 signals coincided with satellite arrays. The genomic coordinates align with those recently identified in *Populus bolleana* via CENH3 CUT&Tag [4], suggesting conservation of functional centromere loci within section *Leuce*. Mapping of ultra-long ONT reads to the assembly confirmed that all centromeric satellite arrays are gapless and fully assembled.

To improve gene structural annotation, we adopted a transcript-based strategy that defines exon-intron structures directly from transcript alignments. We curated a comprehensive poplar transcriptomic dataset from the National Center for Biotechnology Information, comprising 393 samples representing diverse genotypes, developmental stages, and tissues (Table S6, Zenodo). The dataset comprises 1946.66 Gb of NGS RNA-seq data, 356.14 Gb of PacBio Iso-Seq libraries, 17.23 Gb of ONT libraries, and 29.49 Gb of PacBio HiFi libraries spanning 14 *Populus* species across all five major sections of the genus (*Aigeiros*, *Tacamahaca*, *Leuce*, *Leucoides*, and *Turanga*). To avoid biases inherent in predicted protein sequences from related species lacking experimental validation, we restricted protein evidence to the manually curated Swiss-Prot database. We performed the annotation using Evidence-based Annotator (EviAnn), prioritizing transcriptomic data as primary evidence. Additionally, we integrated Helixer terrestrial plant model predictions to improve the accuracy of gene structure prediction in complex regions. In total, 36 264 protein-coding genes were annotated in the *P. davidiana* genome. Compared with other poplar assemblies, this assembly includes 403 unique protein-coding genes, most of which are located in pericentromeric regions and tandem repeat arrays. Only 28 of these genes could be assigned to a GO term or matched to a known conserved domain (Table S7, Zenodo), spanning functional categories such as lipid metabolic process, cytoskeleton organization, defense response to other organisms, and protein modification process, with recognized domains belonging to the zinc-dependent metallopeptidase, the Nck-associated protein 1 (NCKAP1), the leucine-rich repeat receptor-like protein kinase (PLN00113), and the protein kinase catalytic (PKc-like) superfamilies. BUSCO assessment of the *P. davidiana* proteome indicates 99.7% completeness, with only seven missing orthologs. We validated these absences by querying the missing sequences against raw HiFi and ONT reads, finding no alignments. A comparison across other poplar genomes showed that six of these genes are consistently absent. The remaining BUSCO (1353249at3193) is present only in genomes outside section *Leuce*. These results suggest that these genes may be absent from the *P. davidiana* genome (Fig. 1f).

This assembly fully resolves all centromeric satellite arrays, segmental duplications, and 45S rDNA loci, revealing structural variation absent or collapsed in previous poplar assemblies, particularly in pericentromeric and rDNA regions. Evolutionarily, resolving these complex elements enables direct species comparisons of repeat turnover, centromere evolution and rDNA copy-number dynamics within the *Populus* genus. This assembly also provides a complete reference for trait locus mapping, pangenome construction, and poplar breeding.

## Data Availability

The *Populus davidiana* genome assembly, annotations, and supplementary materials have been deposited in Zenodo (DOI: 10.5281/zenodo.18321741) and the CNCB Genome Warehouse (BioProject: PRJCA054038). Raw sequencing data are available in the National Center for Biotechnology Information Sequence Read Archive database under BioProject PRJNA1390897.

## References

[1] Tuskan GA, Difazio S, Jansson S. et al. The Genome of Black Cottonwood, *Populus trichocarpa* (Torr. & Gray). Science. 2006;313:1596–604 10.1126/science.1128691 16973872

[2] Schwacke R, Bolger ME, Usadel B. PubPlant: a continuously updated online resource for sequenced and published plant genomes. Front Plant Sci. 2025;16:1603547 10.3389/fpls.2025.1603547 PMC12235464 40630734

[3] Shen T, Ning Y, Wang Y. et al. Haplotype-resolved telomere-to-telomere genome assembly of *Populus lasiocarpa* unveils retrotransposon-driven centromere evolution. Plant J. 2025;123:e70504 10.1111/tpj.70504 41016064

[4] Wang Y, Zhao L, Wang D. et al. Four near-complete genome assemblies reveal the landscape and evolution of centromeres in Salicaceae. Genome Biol. 2025;26:111 10.1186/s13059-025-03578-7 PMC12046899 40317068

[5] Bi C, Sun N, Hou Z. et al. A gap-free reference genome of *Populus deltoides* provides insights into karyotype evolution of Salicaceae. BMC Biol. 2025;23:201 10.1186/s12915-025-02304-w PMC12232824 40619359

[6] Liu W, Liu C, Chen S. et al. A nearly gapless, highly contiguous reference genome for a doubled haploid line of *Populus ussuriensis*, enabling advanced genomic studies. *For Res (Fayettev)*. 2024;4:e019 10.48130/forres-0024-0016 PMC11524312 39524412

